# microRNA-377 Signaling Modulates Anticancer Drug-Induced Cardiotoxicity in Mice

**DOI:** 10.3389/fcvm.2021.737826

**Published:** 2021-08-16

**Authors:** John Henderson, Praveen K. Dubey, Mallikarjun Patil, Sarojini Singh, Shubham Dubey, Rajasekaran Namakkal Soorappan, Ramaswamy Kannappan, Palaniappan Sethu, Gangjian Qin, Jianyi Zhang, Prasanna Krishnamurthy

**Affiliations:** ^1^Department of Biomedical Engineering, Schools of Medicine and Engineering, University of Alabama at Birmingham, Birmingham, AL, United States; ^2^Division of Molecular & Cellular Pathology, Department of Pathology, School of Medicine, University of Alabama at Birmingham, Birmingham, AL, United States; ^3^Division of Cardiovascular Disease, School of Medicine, University of Alabama at Birmingham, Birmingham, AL, United States

**Keywords:** chemotherapy, cardiotoxicity, LV dysfunction, RNA sequencing, anthracycline, doxorubicin, microRNA

## Abstract

Doxorubicin (DOX, an anthracycline) is a widely used chemotherapy agent against various forms of cancer; however, it is also known to induce dose-dependent cardiotoxicity leading to adverse complications. Investigating the underlying molecular mechanisms and strategies to limit DOX-induced cardiotoxicity might have potential clinical implications. Our previous study has shown that expression of microRNA-377 (miR-377) increases in cardiomyocytes (CMs) after cardiac ischemia-reperfusion injury in mice, but its specific role in DOX-induced cardiotoxicity has not been elucidated. In the present study, we investigated the effect of anti-miR-377 on DOX-induced cardiac cell death, remodeling, and dysfunction. We evaluated the role of miR-377 in CM apoptosis, its target analysis by RNA sequencing, and we tested the effect of AAV9-anti-miR-377 on DOX-induced cardiotoxicity and mortality. DOX administration in mice increases miR-377 expression in the myocardium. miR-377 inhibition in cardiomyocyte cell line protects against DOX-induced cell death and oxidative stress. Furthermore, RNA sequencing and Gene Ontology (GO) analysis revealed alterations in a number of cell death/survival genes. Intriguingly, we observed accelerated mortality and enhanced myocardial remodeling in the mice pretreated with AAV9-anti-miR-377 followed by DOX administration as compared to the AAV9-scrambled-control-pretreated mice. Taken together, our data suggest that *in vitro* miR-377 inhibition protects against DOX-induced cardiomyocyte cell death. On the contrary, *in vivo* administration of AAV9-anti-miR-377 increases mortality in DOX-treated mice.

## Introduction

Cancer is one of the leading causes of death, both in the United States and worldwide (1). Among many available cancer therapies such as surgery, radiation, and immunotherapy, chemotherapy is one of the most powerful tools currently available for the efficient treatment of cancer (2). Chemotherapy drugs act on various cellular signaling pathways but generally are shown to inhibit the cell cycle and induce cell death in proliferating cells through direct or indirect damage to DNA or RNA precursors (3, 4).

Doxorubicin (DOX), a cytotoxic anthracycline antibiotic with antineoplastic activity, is one of the most effective and widely used chemotherapy drugs on the market, yet has a significant side effect of dosage-dependent cardiotoxicity (5–7). Previous studies have shown that both acute and chronic toxicity can induce heart failure through loss of functional cardiomyocytes (5–8). However, the mechanism of cardiac dysfunction during chemotherapy or the susceptibility of patients to develop cardiotoxicity is still elusive.

In this study, we explore the role of microRNA-377 (miR-377) in the regulation of DOX-induced cardiac toxicity and whether modulating miR-377 alters cardiac pathology and dysfunction in DOX-administered mice. microRNAs (miRs) are small non-coding RNAs that are shown to bind to complementary sequences in 3'-UTR of target mRNAs and regulate gene expression. miRs regulate different cellular processes such as proliferation, lineage differentiation, cell metabolism, apoptosis and angiogenesis under both physiological and pathological conditions (9–11). miRs have been implicated in numerous cardiac pathologies, including DOX-induced cardiomyopathy (5, 8, 12–15). From our previous studies, we have shown that miR-377 is significantly upregulated in failing human myocardial tissue, and was specifically upregulated in cardiomyocyte cells following ischemia-reperfusion injury in mouse (16). Also, other reports have shown that miR-377 regulates several pathophysiological conditions, such as diabetic nephropathy (17), oxidative stress (18), premature senescence (19), and cancer (20–23). In this study, we evaluate the effect of anti-miR-377 on DOX-stimulated cardiomyocyte cell death (*in vitro*), and mortality and left ventricular dysfunction after DOX administration in mice.

## Materials and Methods

### Vertebrate Animals

All animal experiments conform to the protocols approved by the Institutional Animal Care and Use Committee (IACUC) at the University of Alabama at Birmingham, USA. Eight-weeks-old C57BL/6J were procured from Jackson Laboratory (Bar Harbor, ME). Before subjecting to experimentation, mice were allowed to acclimatize for 10 days with access to *ad libitum* feed and water.

### Cell Culture, miR-377 Inhibitor (Anti-miR-377) Transfection, and Doxorubicin Stimulation

Human ventricular cardiomyocyte cell line (AC16; Cat# SCC109, MilliporeSigma) were cultured in DMEM/F12 (Cat# D6434, Sigma-Aldrich) containing 2 mm L-Glutamine (Cat# TMS-002-C EMD Millipore), 12.5% fetal bovine serum (FBS; Cat# ES-009-B, EMD Millipore) and 1% Penicillin-Streptomycin Solution (Cat# 15140122, Thermo Fisher Scientific) and incubated in a humidified chamber at 37°C with 5% CO_2_. Cells were seeded in 6-well plate and when 80% confluent, cells were transfected for 48 h with either mirVana^®^ miR-377 inhibitor (30 pMole; Cat# 4464084, Thermo Fisher Scientific) or scrambled inhibitor control (30 pMole; mirVana^®^ miR inhibitor, negative control, Cat# 4464076, Thermo Fisher Scientific) using Lipofectamine RNAi-MAX transfection reagent (Cat# 13778150, Thermo Fisher Scientific) according to the manufacturer's instructions. After transfection, cells were treated for 24 h with doxorubicin (DOX; 1 μm, Cat# D4193, TCI America). Samples were collected and stored at −80°C until further use.

### Real-Time qPCR Analysis

miRs were extracted from cells or tissues using a miReasy kit (Cat# 217004, Qiagen), according to the manufacturers' instructions and our previous studies (16). microRNAs were reverse transcribed using TaqMan™ Advanced miR cDNA Synthesis Kit miR (Cat# A28007, Applied Biosystems™, Thermo Fisher Scientific) using TaqMan Advanced miR Assays for mice and human miR-377 (Cat# 4427975, Thermo Fisher Scientific) according to the manufacturer's instructions. Quantitative real-time PCR was performed in QuantStudio 3 system (Applied Biosystems, Thermo Fisher Scientific) using TaqMan™ Fast Advanced Master Mix (Cat# 4444557, Thermo Fisher Scientific). miR expression was normalized to U6 gene (Cat# 4427975, Thermo Fisher Scientific) and data was represented as fold-change vs. respective control. Total RNA from AC16 cells and mice heart was extracted using a RNeasy mini kit (Cat# 74106, Qiagen) as per the manufacturers' instructions. The RNA was reverse transcribed to cDNA using RevertAid First Strand cDNA Synthesis Kit (Cat# K1622, Thermo Fisher Scientific). Relative gene expression was assessed by qPCR using the PowerUp™ SYBR™ Green Master Mix (Cat # A25778, Thermo Fisher Scientific) using gene specific primers. Expression of the target genes was normalized to housekeeping genes (GAPDH, β-Tubulin, or 18S rRNA) and was represented as fold change vs. control. All primer sequences used in the study are available in Supplementary Table 1.

### Assessment of Apoptotic Cell Death

Human ventricular cardiomyocytes (AC16) were plated on chamber slides and transfected with miR-377 inhibitor (anti-miR-377) or scramble-anti-miR negative control (Scr) for 48 h followed by stimulation with doxorubicin (1 μm) for 24 h. DOX-induced apoptosis was evaluated using Click-iT Plus TUNEL assay kit (Cat# C10618, Invitrogen, Thermo Fisher Scientific) and DNA was stained with Hoechst-33342 as per the manufacturer's instructions. Stained cells were imaged using fluorescence microscopy (IX83, Olympus). Images were analyzed using ImageJ (NIH) software and percent TUNEL-positive cells were counted.

### RNA Sequencing and Bioinformatics Analysis

AC16 cells were cultured and transfected either with miR-377 inhibitor (anti-miR-377) or scramble-anti-miR negative control (scramble control), followed by DOX (1 μm) treatment for 24 h (*n* = 2 for each group). Total RNA was isolated using an RNAeasy mini kit (Cat#74106, Qiagen) according to the manufacturer's instructions. RNA sequencing and bioinformatics analyses were performed by Genewiz Inc. (South Plainfield, NJ). Briefly, RNA samples were quantified, and RNA integrity was assessed using Agilent TapeStation (Agilent Technologies, USA). RNA integrity number (RIN) for all the samples was ~10. RNA sequencing libraries were constructed using NEBNext^®^ Ultra™ RNA Library Prep Kit for Illumina^®^ (NEB, USA) according to the manufacturer's instructions. RNA sequencing was performed on the Illumina HiSeq^®^ using a 2 × 150 bp paired-end configuration. Raw sequence data (fastq files) were trimmed using Trimmomatic v.0.36. The trimmed reads were mapped to the *Homo sapiens* GRCm38 (available on ENSEMBL) using the STAR aligner v.2.5.2b. Unique gene hit counts were calculated using the Subread package v.1.5.2. and the unique reads that fell within exon regions were counted. Next, differentially expressed genes (DEGs) were identified between anti-miR-377+DOX and (*n* = 2) and scrambled control+DOX (*n* = 2) using DESeq2 package. The Wald test was used to calculate *p-*values and log2 fold changes (log2FC). Benjamini-Hochberg test was performed to calculate adjusted *p-*values. Genes with adjusted *p-*values < 0.05 and absolute log2FC > 1 were considered differentially expressed genes (DEGs).

For Gene ontology analysis, differentially expressed genes were clustered by their gene ontology, and the enrichment of gene ontology terms was tested using Fisher exact test (GeneSCF v1.1-p2). The pathways with an adjusted *p-*value < 0.05 were considered significantly enriched.

### *In vivo* Adenovirus (AAV9)-Mediated miR-377 Inhibition and Doxorubicin Administration in Mouse

Adeno-associated virus, serotype 9 (AAV9)-based murine shRNA-miR-377 and scrambled-negative control under a cytomegalovirus (CMV) promoter was procured from Vector Biolabs (Malvern, Pennsylvania). The construct was then packaged into AAV-9 by transfection of HEK293 cells, and viral particles were purified by CsCl_2_ centrifugation (Vector Biolabs). Each mouse was intravenously injected (*via* tail vein) with either 200 μl of AAV9-shRNA-miR-377 (AAV9-Anti-miR-377; ~8.75 × 10^11^ particles/mice; *n* = 12) or a scrambled AAV9 control (AAV9-scrambled control; ~8.75 × 10^11^ particles/mice; *n* = 12). After 28 days following AAV9 infection, miR-377 inhibition in left ventricle was confirmed by qRT-PCR (*n* = 2 in each group), and mice were administered DOX (20 mg/kg diluted in PBS; *n* = 10 in each group) *via* intraperitoneal (i.p.) injection. A subset of mice (*n* = 3 in each group) was sacrificed at day 3 for target gene expression analyses. The remaining seven mice in each group were used for survival study. Since there was higher mortality in AAV9-anti-miR-377/DOX-administered group (only 2 mice survived), the experiment was terminated on day 6 after DOX administration.

### Echocardiography

Transthoracic two-dimensional M-mode echocardiography was performed using the Vevo2100 (VisualSonics, Toronto, Canada) equipped with a 30-MHz transducer as described previously (16). The mice were anesthetized with a mixture of 1.5% isoflurane and oxygen (1 L/min). Echocardiography was recorded at baseline (prior to AAV9 administration), 28 days following AAV9 administration, and on day 6 after DOX administration. B-Mode and M-Mode tracings were used to measure left ventricle (LV) percent fractional shortening (%FS), wall thickness, cardiac output, and percent ejection fraction (%EF) as described earlier (16).

### Histological Analysis

Mouse hearts were quickly excised after euthanasia and fixed in 10% neutral-buffered formalin solution. Paraffin-embedded tissue sections (5 μm in thickness) were stained with hematoxylin and eosin and viewed under the microscope. The cross-sectional area of cardiomyocytes was measured using ImageJ (NIH) from captured images.

### Statistical Analyses

Data were analyzed using two-tailed unpaired *t*-test or one-way ANOVA with Dunnet correction. All values are presented as mean ± SEM. Probability (*P*) values of ≤0.05 were considered statistically significant.

## Results

### Doxorubicin Upregulates miR-377 Expression in Mouse Left Ventricles and Human Ventricular Cardiomyocytes (AC16)

**Figure 1:**
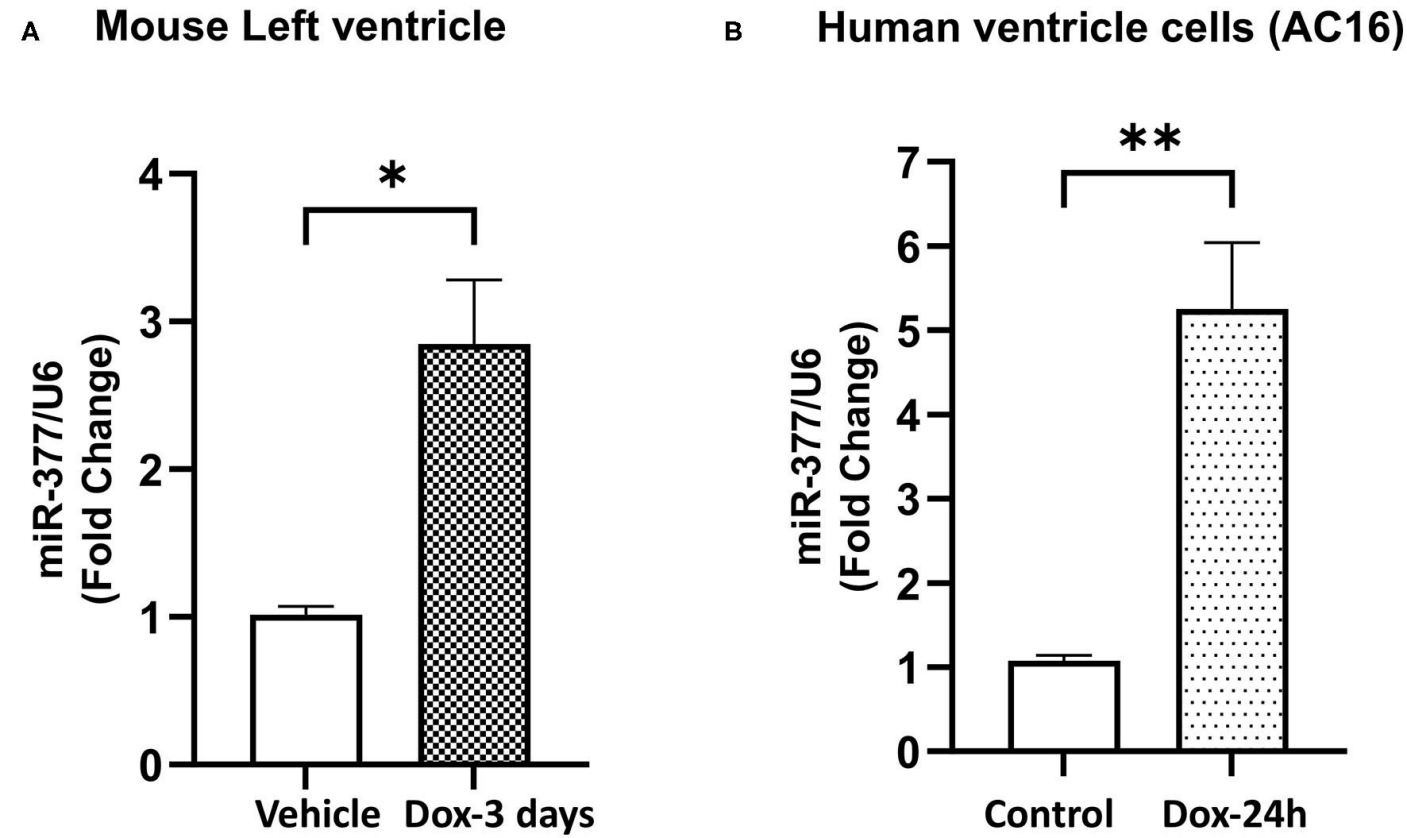
Doxorubicin upregulates miR-377 expression in mouse myocardium and human ventricular cardiomyocyte cell (AC16). **(A)** Quantification of miR-377 expression in mouse myocardium following 20 mg/kg DOX treatment for 3 days. miR-377 expression was normalized to U6 and values are shown as fold change. Data are represented as mean ± SEM (*n* = 3, *p < 0.05 vs. vehicle control). **(B)** Quantification of miR-377 expression in human ventricular cardiomyocytes (AC16) following 1 μm DOX treatment for 24 h. miR-377 expression was normalized to U6 and values are shown as fold change. Data are represented as mean ± SEM (*n* = 3, **p < 0.01 vs. untreated control cells).

Our previous study has showed that miR-377 expression was significantly increased in cardiomyocytes (as compared to other cell types) following ischemia-reperfusion injury in mouse (16). To study the effect of doxorubicin on miR-377 expression, we administered DOX (20 mg/kg; i.p.) in mice. After 3 days, we evaluated miR-377 expression in the left ventricles. We observed a significant increase in myocardial miR-377 expression in DOX-administered mice, compared to the vehicle control group (Figure 1A, *p* = 0.0138). Furthermore, human ventricular cardiomyocytes (AC16 cells) were treated with 1 μm DOX for 24 h and miR-377 expression was quantified by RT-qPCR. We observed that doxorubicin exposure significantly increased miR-377 expression in cardiomyocytes as compared to the untreated control cells (Figure 1B, *p* = 0.0015).

### Inhibition of miR-377 Attenuates DOX-Induced Cardiomyocyte Cell Death and Oxidative Stress-Related Gene Expression

**Figure 2:**
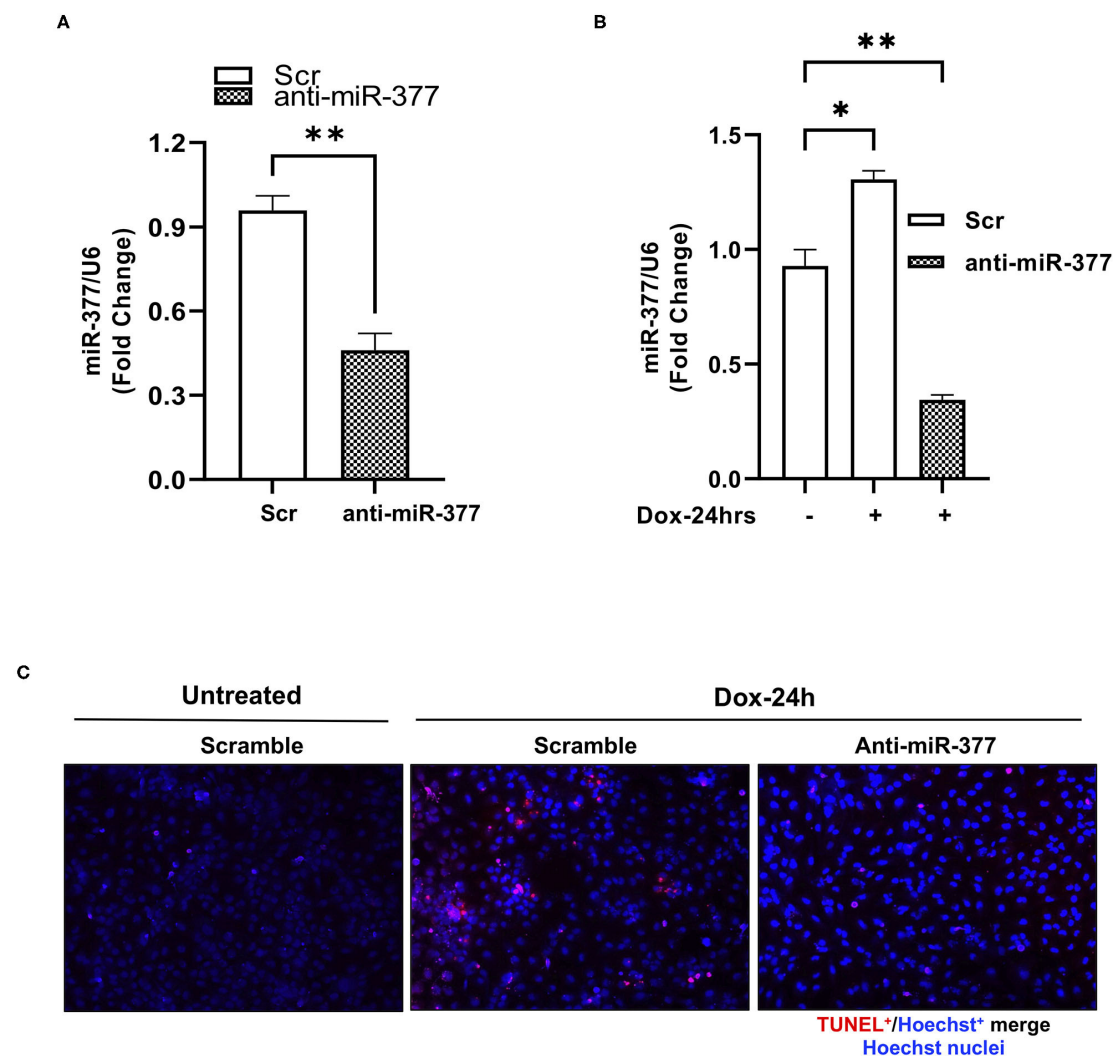
Inhibition of miR-377 attenuates DOX-induced cardiomyocyte cell death. **(A)** Evaluation of transfection efficiency of miR-377 inhibitor in AC16 cell was performed by qPCR. Quantification of miR-377 expression in AC16 cells transfected with either anti-miR negative control (Scramble/Scr) or miR-377 inhibitor (anti-miR-377). miR-377 expression was normalized to U6 and values are shown as fold change. Data are represented as mean ± SEM (*n* = 3, ***p < 0.001 vs. scrambled control cells), **(B)** Quantification of miR-377 expression in AC16 cells transfected with either anti-miR-377 or Scramble, following 1 μm DOX treatment for 24 h. miR-377 expression was normalized to U6 and values are shown as fold change. Data are represented as mean ± SEM (*n* = 3, *p < 0.05, **p < 0.01 vs. scrambled control cells). **(C)** Representative image of TUNEL assay in AC16 cells transfected with scramble control without DOX, scramble control with DOX, and anti-miR-377 with DOX; TUNEL-positive nuclei (red) and nuclear label Hoechst (blue).

Chemotherapy-induced cardiotoxicity is associated with the generation of reactive oxygen species (ROS) and cell death (24, 25). Also, in our previous study, we demonstrated that transplantation of miR-377-silenced human CD34^+^ cells attenuated myocardial ischemia-reperfusion injury-induced interstitial fibrosis and left ventricular dysfunction in mice (16). To explore the role of miR-377 in DOX-induced cell death and oxidative stress in cardiomyocytes, AC16 cells were transfected with either anti-miR-377 or non-specific miR inhibitor negative control (scrambled control). Following transfection, cells were treated with 1 μm DOX for 24 h. Apoptosis was assessed by TUNEL assay and expression of antioxidant genes was assessed by RT-qPCR. The transfection efficiency of anti-miR-377 was confirmed by qPCR in AC16 cells, transfected with either anti-miR-377 or scramble control (Figure 2A). Figure 2B shows that DOX-mediated miR-377 induction was inhibited in AC16 cells after anti-miR-377 treatment. Furthermore, DOX treatment increased apoptosis in cardiomyocytes when compared to the untreated scrambled control cells. Interestingly, miR-377 inhibition significantly attenuated DOX-induced apoptosis as compared to DOX-treated scrambled control cells (Figure 2C). Furthermore, mRNA expression analysis for antioxidant genes showed that miR-377 inhibition upregulates the expression of antioxidant genes, such as GCLC, NQO1, SOD1, etc., (Supplementary Figure 1).

### Transcriptome Analysis Showed a Distinct Gene Expression Profile in DOX-Stimulated Cardiomyocytes (AC16 Cells) After miR-377 Inhibition

**Figure 3:**
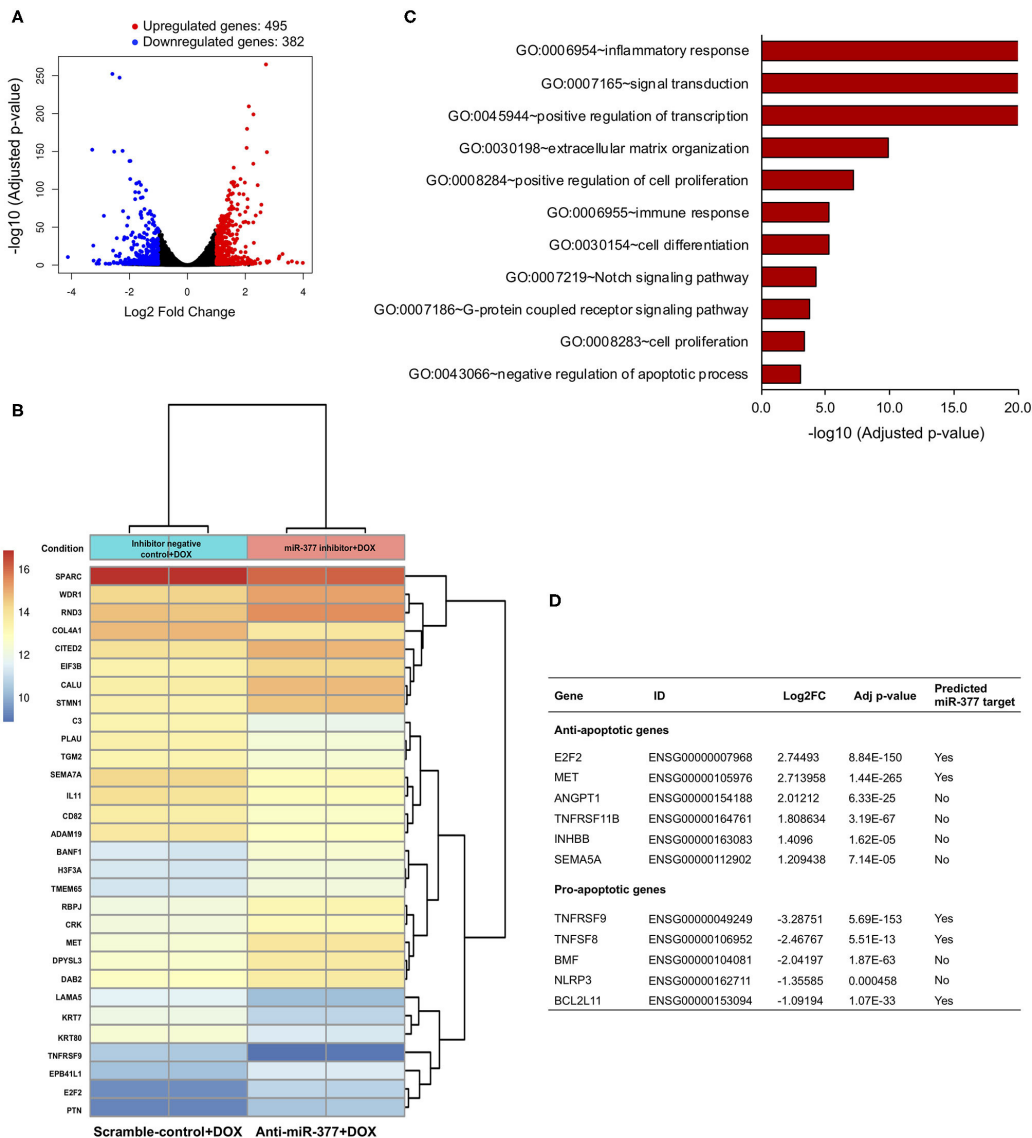
RNA sequencing and gene ontology analysis to show the effect of miR-377 inhibition on the transcriptome of DOX-stimulated AC16 cardiomyocytes. **(A)** The global transcriptional change across the compared groups was visualized by a volcano plot, with log2 fold change of each gene is represented on the -axis and the log10 of its adjusted *p-*value is on the y-axis. Genes with an adjusted *p-*value < 0.05 and a log2 fold change ≥1 are considered upregulated and indicated by red dots, whereas genes with an adjusted *p-*value < 0.05 and a log2 fold change ≤ -1 are considered downregulated and indicated by blue dots. **(B)** A bi-clustering heatmap representing the expression profile of the top 30 differentially expressed genes sorted by their adjusted *p-*value by plotting their log2 transformed expression values in between anti-miR-377 + DOX (*n* = 2) and anti-miR negative control+DOX (scrambled control+DOX, *n* = 2) groups. **(C)** GO analysis: Gene ontology terms that are significantly enriched with an adjusted *p-*value < 0.05 in the differentially expressed genes. **(D)** List of cell survival genes that were differentially expressed in DOX-stimulated anti-miR377-treated cells. The list also shows if the differentially expressed genes are predicted targets of miR-377.

miRs bind to a complementary sequence on 3'-UTR of a number of target mRNAs and regulate their expression, thus playing an important regulatory role in numerous signaling pathways (26). To elucidate the effect of miR-377 inhibition during DOX-induced cardiotoxicity, AC16 cells were transfected with either anti-miR-377 or miR inhibitor negative control (scramble), and treated with DOX for 24 h. As an unbiased approach, we performed RNA-sequencing (RNA-seq) and Gene Ontology (GO) enrichment analyses to identify transcriptome-wide signaling pathways that are differentially altered. The overall similarity among samples was assessed by the Euclidean distance between samples (Supplementary Figure 2A). RNA-seq analysis showed a total of 877 differentially expressed genes between the two groups. The global transcriptional change between the groups (anti-miR-377+DOX and scramble control+DOX) is represented as a volcano plot with 495 significantly upregulated genes (red dots, Figure 3A) and 382 significantly downregulated genes (blue dots, Figure 3A). The complete list of significant, differentially expressed genes is submitted as Supplementary Data Sheet 1. A heatmap representing the hierarchical clustering of the top 30 differentially expressed genes (DEGs) among the samples is represented in Figure 3B. Further analyses of DEGs showed differential expression of genes involved in apoptosis regulation, TNF signaling, cell survival, and cell growth and proliferation.

Interestingly, GO analyses further revealed significant changes in several signaling pathways involved in inflammation, cell proliferation and differentiation, integrin, and GPCR signaling (Figure 3C and Supplementary Figure 2B). In addition, we found that apoptosis was also one of the significantly altered biological processes between the groups. We identified a number of DEGs that play a role in cell death. With the inhibition of miR-377, several anti-apoptotic genes were significantly upregulated, while many pro-apoptotic genes were downregulated (Figure 3D). Next, we validated the expression of some of these cell survival genes in AC16 cells (scramble+DOX and anti-miR-377+DOX) by qRT-PCR. We observed that the expression of anti-apoptotic gene E2F2 was elevated, whereas pro-apoptotic genes, such as TNF Receptor Superfamily Member 9 (TNFSFRF9), Bcl_2_-modifying factor 2 (BMF), and TNF Superfamily Member 8 (TNFSF8) were suppressed in anti-miR-377-transfected cells after doxorubicin stimulation (Supplementary Figures 3A–D). Moreover, hypertrophy-associated markers, like Atrial natriuretic peptide (ANP) and Myosin Heavy Chain 7 (MYH7) were also downregulated (Supplementary Figures 3E,F).

### Mice Receiving AAV9-Anti-miR-377 Shows Increased Mortality Following Doxorubicin Administration

**Figure 4:**
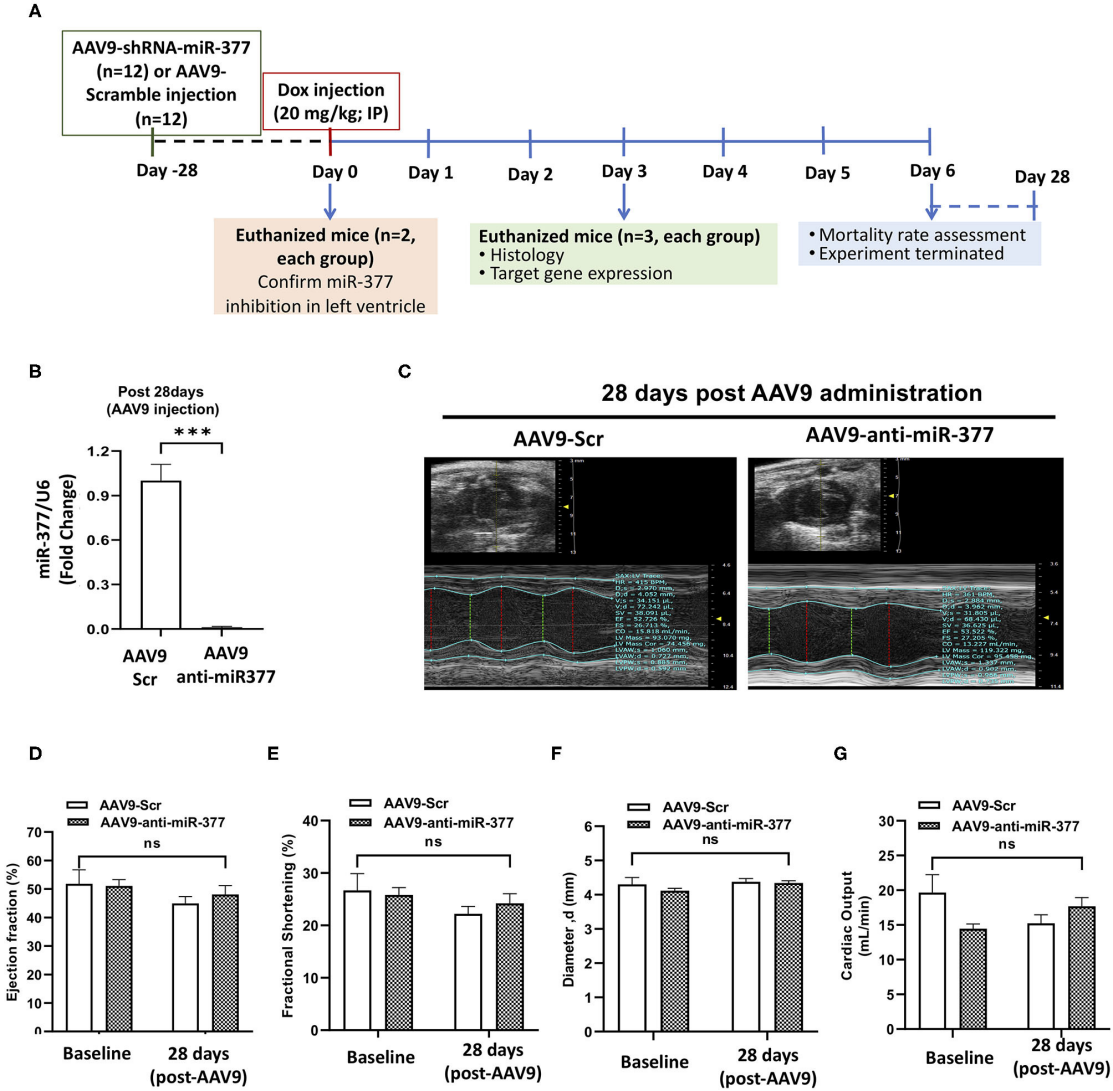
AAV9-based inhibition of miR-377 in mouse myocardium and its effect on LV function **(A)** *in vivo* study design. **(B)** Quantification of miR-377 expression in the mouse myocardium at 28 days after AAV9 treatment. miR-377 expression was normalized to U6 and values are shown as fold change. Data are represented as mean ± SEM (*n* = 2/group). **(C)** Representative echocardiography m-mode tracings 28 days post AAV9 administration. **(D)** Percent ejection fraction, **(E)** percent fractional shortening, **(F)** Left ventricular diameter at diastole, and **(G)** cardiac output of AAV9-Scr (anti-miR) and AAV9-anti-miR377 mice at the baseline and 28 days post-AAV9 administration. Data are represented as mean ± SEM (*n* = 10/group).

**Figure 5:**
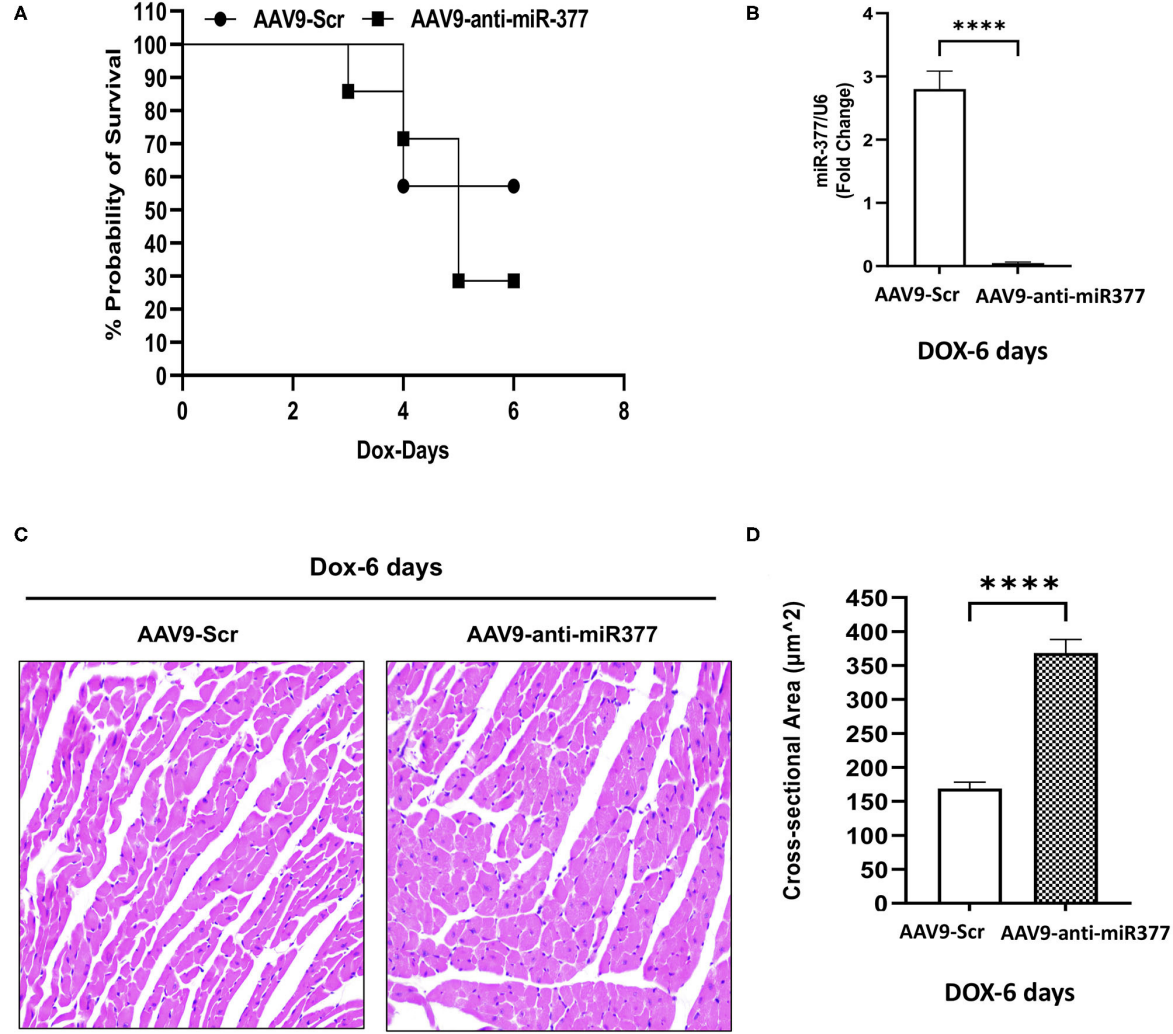
AAV9-anti-miR-377 injection in mice accelerates mortality following DOX administration and is associated with changes in gene expression. **(A)** Kaplan-Meier survival curve to show mortality in the mice pretreated with AAV9-scramble (anti-miR) control or AAV9-anti-miR-377 followed by DOX (20 mg/kg) administration (*n* = 10/group). **(B)** Quantification of miR-377 expression in the mouse myocardium of AAV9-scramble (anti-miR) control or AAV9-anti-miR-377 at 28 days after AAV9 treatment. miR-377 expression was normalized to U6 and values are shown as fold change. Data are represented as mean ± SEM (*n* = 3/group, ****p < 0.0001). **(C)** Representative Hematoxylin & Eosin-stained sections of left ventricle of AAV9-scramble-control or AAV9-anti-miR-377 at day 6. **(D)** Mean cross-sectional area of cardiomyocytes in the left ventricles of AAV9-scramble-control or AAV9-anti-miR-377 mice.

Our *in vitro* studies suggested that miR-377 protects against DOX-induced cell death and oxidative stress. To determine the biological significance of the above effects, we blocked miR-377 (by pretreating the mice with AAV9-shRNA-miR-377) and evaluated its effect on doxorubicin-induced cardiotoxicity and mortality (experimental design shown in Figure 4A). Following 28 days of AAV9 injection (before DOX administration), miR-377 knockdown in the myocardium was confirmed by quantitative qRT-PCR (Figure 4B). Furthermore, left ventricular function at baseline (before AAV9 injection) and at 28 days post-AAV9 injection was assessed by echocardiography (Figure 4C). Echocardiography analyses showed no significant changes in %EF, %FS, and LV chamber diameter at diastole, and cardiac output (Figures 4D–G) between AAV9-anti-miR-377 and AAV9-scrambled control (AAV9-Scr) administered groups at 28 days post-AAV9 injection (before DOX administration). Similar trend was observed in other echocardiographic parameters (Supplementary Figures 4A–D). However, contrary to *in vitro* effects, upon DOX-administration, mortality was expedited and higher in AAV9-anti-miR-377 group (only two mice survived), when compared to AAV9-scrambled control group (Figure 5A; survival curve).

### *In vivo* Inhibition of miR-377 Followed by DOX Administration Leads to Increased Cardiomyocyte Size and Changes in Gene Expression Related to Cardiac Remodeling

**Figure 6:**
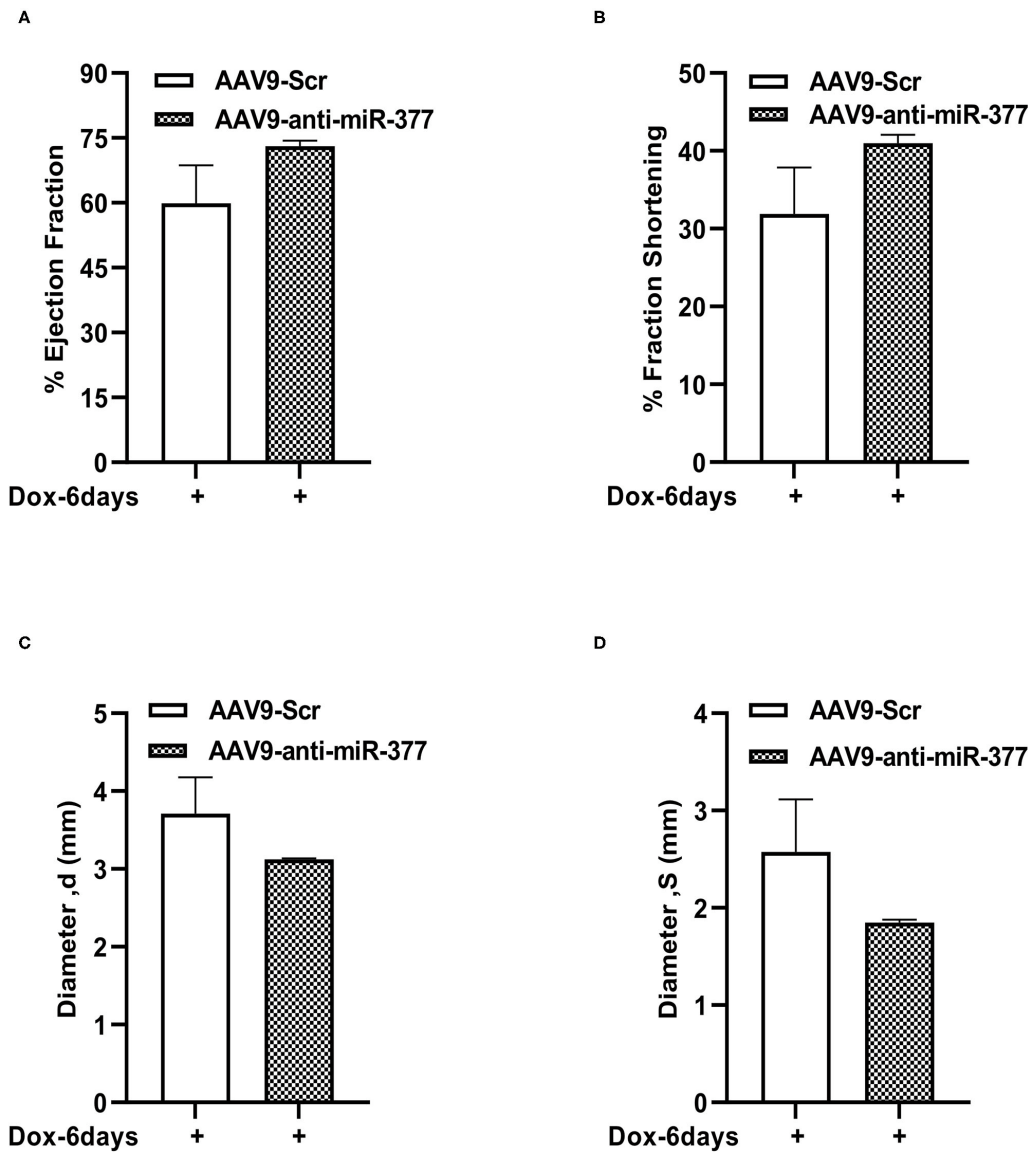
Left ventricular function assessment in mice with AAV9-anti-miR-377 injection, 6 days after DOX administration. **(A)** Percent ejection fraction, **(B)** percent fractional shortening, **(C)** Left ventricular diameter at diastole, and **(D)** Left ventricular diameter at systole of AAV9-Scr (anti-miR) and AAV9-anti-miR377 mice after 6 days of DOX stimulation. Data are represented as mean ± SEM (*n* = 3 in AAV9-Scr and *n* = 2 in AAV9-anti-miR-377).

Our mouse data showed increased mortality in AAV9-anti-miR-377 mice following doxorubicin administration. Therefore, we asked if AAV9-anti-miR-377 had any effect on cardiac remodeling and cardiac function after DOX administration. We validated miR-377 knockdown in the myocardium at 6 days after DOX administration by quantitative qRT-PCR (Figure 5B, *p* < 0.0001). Histological analysis (Hematoxylin and Eosin staining) of the heart tissue of the mice (that survived on day 6) showed an increase in the cross-sectional area of individual cardiomyocytes in the left ventricles (Figures 5C,D). Interestingly, echocardiographic analyses at day 6 after DOX administration, although, not significant, revealed a trend toward higher %EF and % FS, and decreased LV diameters in AAV9-anti-miR-377 group as compared to the AAV9-scrambled control group (Figures 6A–D). Furthermore, anti-miR-377 mice after DOX administration showed changes in several genes related to cardiac remodeling and pathology, such as Myosin Heavy chain 6 (MYH6), MYH7, Natriuretic Peptide A (NPPA), Growth Differentiation Factor 15 (GDF15), and MET Proto-Oncogene, Receptor Tyrosine Kinase (MET) in left ventricular tissues (Supplementary Figures 5A–F).

## Discussion

**Figure 7:**
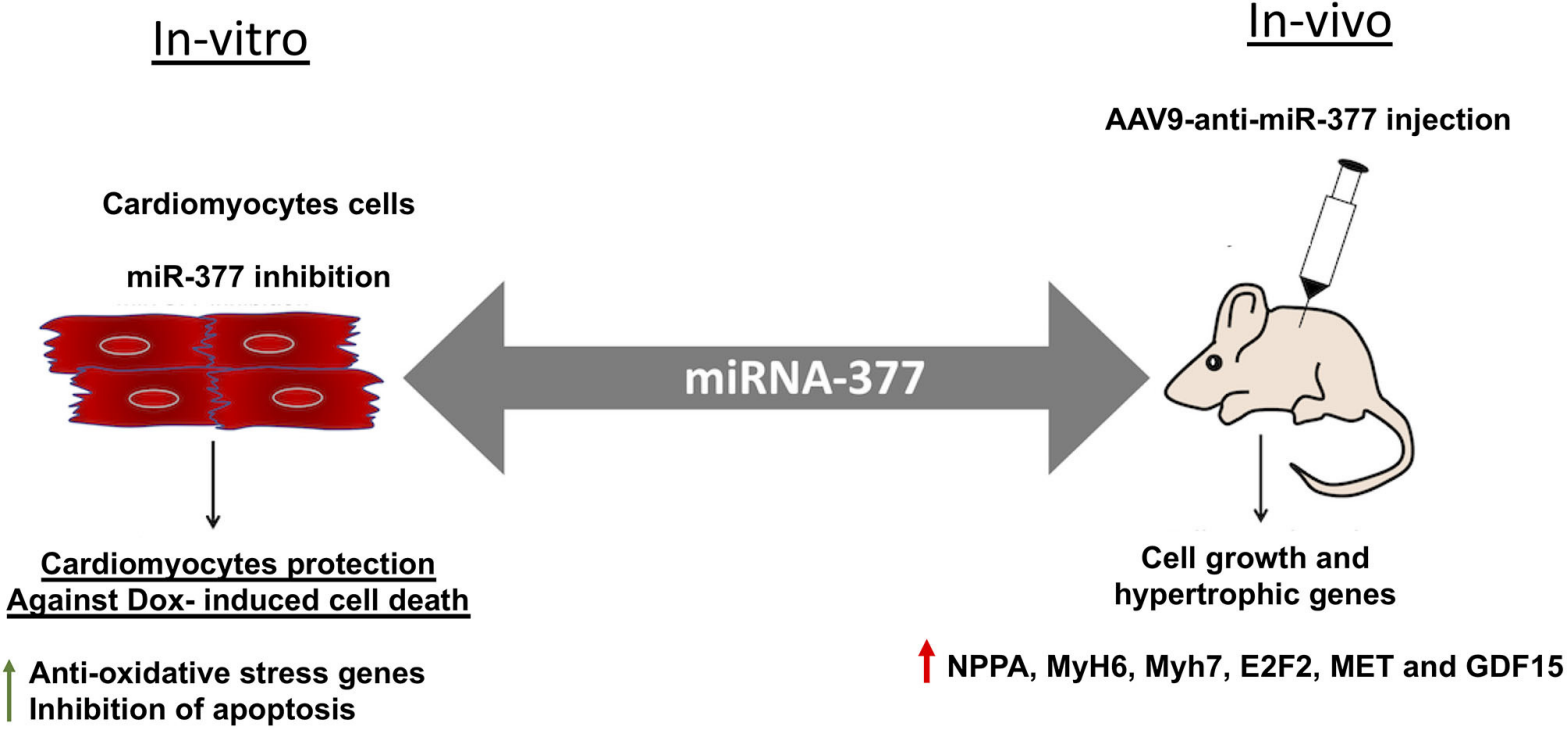
Graphical representation showing the effect of miR-377 inhibition *in-vitro* and *in-vivo* on DOX-induced cardiotoxicity. *In-vitro*, miR-377 inhibition leads to upregulation of anti-oxidative stress response genes and therefore, inhibition of DOX-induced cardiomyocyte apoptosis. On the contrary, *in-vivo* inhibition of miR-377 (using AAV9-anti-miR-377) leads to increased mortality, potentially due to accelerated cell growth and upregulation of genes such as MET, E2F2, and GDF15 (that are involved in adverse cardiac remodeling).

Doxorubicin, (an anthracycline) is still the chemotherapeutic drug of choice for treating many cancers; however, is well-known for its significant side effect of dose-dependent cardiotoxicity (5, 7, 8). While the mechanisms of action are well-studied and evolving, strategy for preventing and managing chemotherapy-induced cardiotoxicity remains unknown. Our previous study has shown that cardiac stress and damage increases miR-377 expression in the mouse heart and was specifically higher (~20-fold) in cardiomyocytes (16). The present study is aimed to explore the potential role of miR-377 in the progression of DOX-induced cardiac pathology and dysfunction, and whether inhibiting miR-377 limits DOX-induced cardiotoxicity. Our data suggest that miR-377 inhibition protects against DOX-induced cardiomyocyte cell death *in vitro*, whereas AAV9-based anti-miR-377 accelerated mortality in mice after DOX-administration (graphical representation is shown in Figure 7).

We first examined the effect of DOX on miR-377 expression in ventricular cardiomyocytes (AC16) and in the myocardium of mouse administered with DOX. We found that DOX treatment induced a significant increase in miR-377 in both human AC16 cardiomyocytes and in mouse myocardium. This allowed us to speculate that miR-377 may be playing a role in progression of DOX-induced cardiotoxicity. The data also corroborated with our previous study showing increased expression of miR-377 in human failing heart samples (16). Cardiomyocytes have a lower ability to regenerate and are potentially more susceptible to adverse effects of chemotherapy (27–29). DOX has been shown to stimulate ROS production leading to increased cell death in the myocardium. We next evaluated the biological effects of miR-377 on cardiomyocyte. We found that inhibition of miR-377 protects against DOX-induced cardiomyocyte apoptosis *in vitro*. Furthermore, a number of genes involved in oxidative stress, specifically, antioxidant genes were upregulated in anti-miR-377-treated cells. These data suggest that anti-miR-377 might have protective effects against DOX-induced cardiotoxicity *via* protection against cardiomyocyte apoptosis. However, the molecular mechanism is not clear.

microRNAs bind to complementary sequences in the 3'-UTR of target genes, and therefore regulate their expression and function (13, 26, 30, 31). Previous reports, including our study have shown that miR-377-mediated deleterious effects on biological processes were by regulating the expression of a number of target genes, such as STK35, VEGF-A, HO-1, and SOD1/2 (16–18, 32). To determine the target genes of miR-377 in DOX-stimulated cardiomyocytes, we took an unbiased RNA-sequencing approach and evaluated the gene expression profile in human cardiomyocyte cell line (AC16) transfected with either miR-377 inhibitor (anti-miR-377) or miR inhibitor negative control (scrambled control) followed by stimulation with DOX for 24 h. We found that there were significant differences in gene expression patterns between the control and anti-miR-377 groups. When compared to the scramble-control group, there were 495 genes significantly upregulated and 382 genes significantly downregulated in anti-miR-377-treated cells. Furthermore, GO analysis revealed a number of molecular pathways that were significantly altered, including the signaling mechanisms related to inflammatory response, proliferation, cell cycle regulation, and regulation of apoptosis. Further analysis revealed that cell survival genes such as pleiotrophin (PTN), E2F2, MET, and Inhibin, beta B (INHBB) were highly upregulated in anti-miR-377+DOX group as compared to the scramble control+DOX group. Also, we observed that TNF inflammatory response genes and pro-apoptotic genes were downregulated in the anti-miR-377 treated group as compared to scramble-control transfected cells. Taken together, these data suggest that miR-377 inhibition might protect against DOX-induced cell death, possibly through the downregulation of pro-apoptotic genes and upregulation of cell survival genes.

One of the most common manifestations of cardiotoxicity is LV dysfunction. Therefore, we performed *in vivo* experiment to determine the effects of miR-377 inhibition on DOX-induced cardiac pathophysiological effects in mice. Adeno-associated virus 9 (AAV9) was used to deliver anti-miR-377 since AAV9 has been shown to cardiotropic (33). miR-377 inhibition in the myocardium after 28 days following AAV9 administration was confirmed by qRT-PCR. However, echocardiography analyses of left ventricle showed no difference in LV function between baseline (before AAV9 injection) group and after 28 days of AAV9 injection, suggesting AAV9 injection did not have adverse effect on the left ventricular function. Interestingly, following DOX administration, we observed increased mortality in mice receiving anti-miR-377, which was contrary to our *in vitro* data. However, our RNA-seq data identified several genes, including E2F2 and MET that are upregulated in anti-miR-377 group. Interestingly, E2F2 and MET have been implicated in adverse cardiac remodeling (34, 35), suggesting their involvement in adverse outcomes in anti-miR-377 administered group. To this end, our data shows increased expression of MET and E2F2 after Dox treatment in anti-miR-377-administered mice as compared to scramble control group (Supplementary Figure 5). Moreover, we also observed increased expression of cardiac hypertrophic genes, such as MYH6, MYH7, NPPA, and GDF15. GDF15, a cardiac hypertrophy marker, is also reported to be a more sensitive and non-traditional biomarker for doxorubicin-induced cardiotoxicity (36, 37). Collectively, these data suggest that miR-377 inhibition dysregulated the expression of MET and E2F2, post-DOX administration. While not conclusive, these potential signaling pathways could be playing a role in increased toxicity of doxorubicin on cardiomyocytes in anti-miR-377-administered mice. Further investigation into the exact mechanism is warranted.

Echocardiography data analyses of the surviving mice revealed an increasing trend in the ejection fraction and fractional shortening between the groups, while LV diameters were slightly reduced in AAV9-anti-miR-377 group. Furthermore, H&E staining showed increase in cross-sectional area of cardiomyocytes in anti-miR-377 group following DOX administration. While the reason behind the observed phenomenon is not clear, upregulation of cell growth genes, E2F2 and MET in anti-miR-377 could be the plausible mechanisms. Previous study has shown that E2F2 stimulation leads to cardiomyocyte proliferation *in vivo* (38). Investigation of cardiac hypertrophy and other remodeling genes exhibited similar response in anti-miR-377-administered group upon DOX stimulation. These data suggest that miR-377 inhibition either stimulates hypertrophic response or increases cardiac cell growth and proliferation. However, further studies are needed to determine the role of miR-377 in cardiomyocyte growth and proliferation or potential hypertrophic response.

### Study Limitations

Human AC16 cells may not accurately reflect *in vivo* cardiomyocyte behavior due to differences induced in immortalized phenotype. In addition, the absence of underlying comorbid cancer in the current mouse model may not reflect the exact pathophysiological conditions expected to be seen in cancer patients receiving doxorubicin therapy.

## Data Availability Statement

The original contributions presented in the study are publicly available. This data can be found here: https://www.ncbi.nlm.nih.gov/bioproject/PRJNA748761.

## Ethics Statement

The animal study was reviewed and approved by Institutional Animal Care and Use Committee (IACUC) at the University of Alabama at Birmingham (UAB).

## Author Contributions

JH, PK, MP, and PKD conceptualized the research project. JH, MP, and PKD performed the experiments and are the guarantors of this work. JH, PKD, MP, SS, SD, and PK analyzed and organized the data. PK, MP, PKD, RNS, RK, PS, SS, GQ, JZ, and JH interpreted the results. JH, PK, MP, SS, and PKD wrote the manuscript draft All authors have read the manuscript, provided critical appraisal, conceptual insights, discussed the results, interpretation, and commented on the manuscript at all stages.

## Conflict of Interest

The authors declare that the research was conducted in the absence of any commercial or financial relationships that could be construed as a potential conflict of interest.

## Publisher's Note

All claims expressed in this article are solely those of the authors and do not necessarily represent those of their affiliated organizations, or those of the publisher, the editors and the reviewers. Any product that may be evaluated in this article, or claim that may be made by its manufacturer, is not guaranteed or endorsed by the publisher.
