# Supplementary material for: microRNA-377 Signaling Modulates Anticancer Drug-Induced Cardiotoxicity in Mice
Source: Front Cardiovasc Med. 2021 Aug 16;8:737826. doi: 10.3389/fcvm.2021.737826 (PMC8415717; doi:10.3389/fcvm.2021.737826)
Supplement: Supplementary file 2 [file Data_Sheet_2.PDF]

Supplementary Figure S1

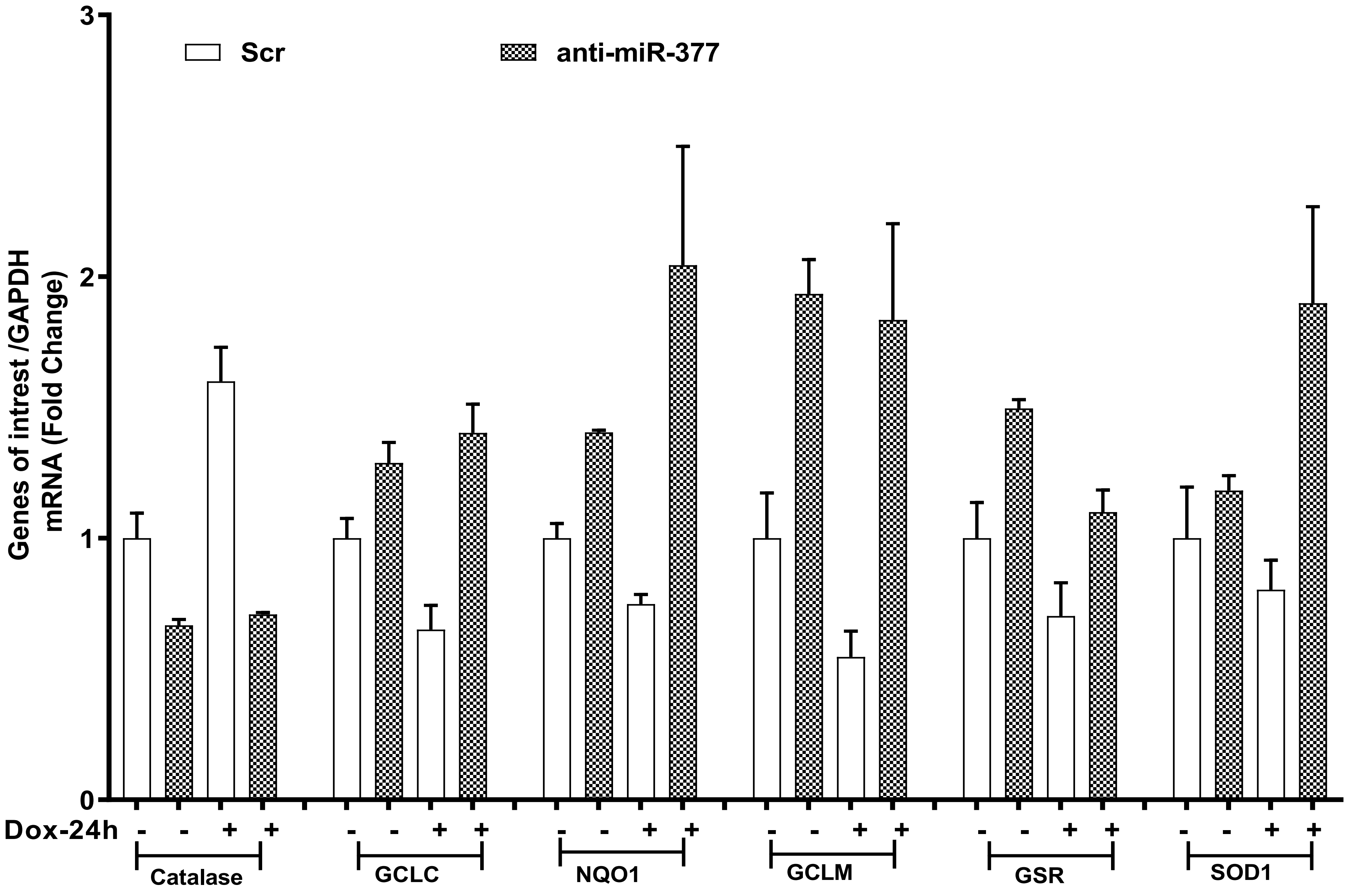

Figure S1. Inhibition of miR-377 augments antioxidant gene expression in DOX-stimulated AC16 cells. Quantification of mRNA expression of antioxidant genes in AC16 cells transfected with either anti-miR-377 or anti-miR negative control (Scr), following 1 μM DOX treatment for 24 hours. mRNA expression of the genes was normalized to GAPDH and values are shown as fold change. Data are represented as mean±SEM. GCLC: Glutamate-Cysteine Ligase Catalytic Subunit; NQO1: NAD(P)H Quinone Dehydrogenase 1; GCLM: Glutamate-Cysteine Ligase Modifier Subunit; GSR: Glutathione-Disulfide Reductase; SOD1: Superoxide Dismutase 1.

Supplementary Figure S2

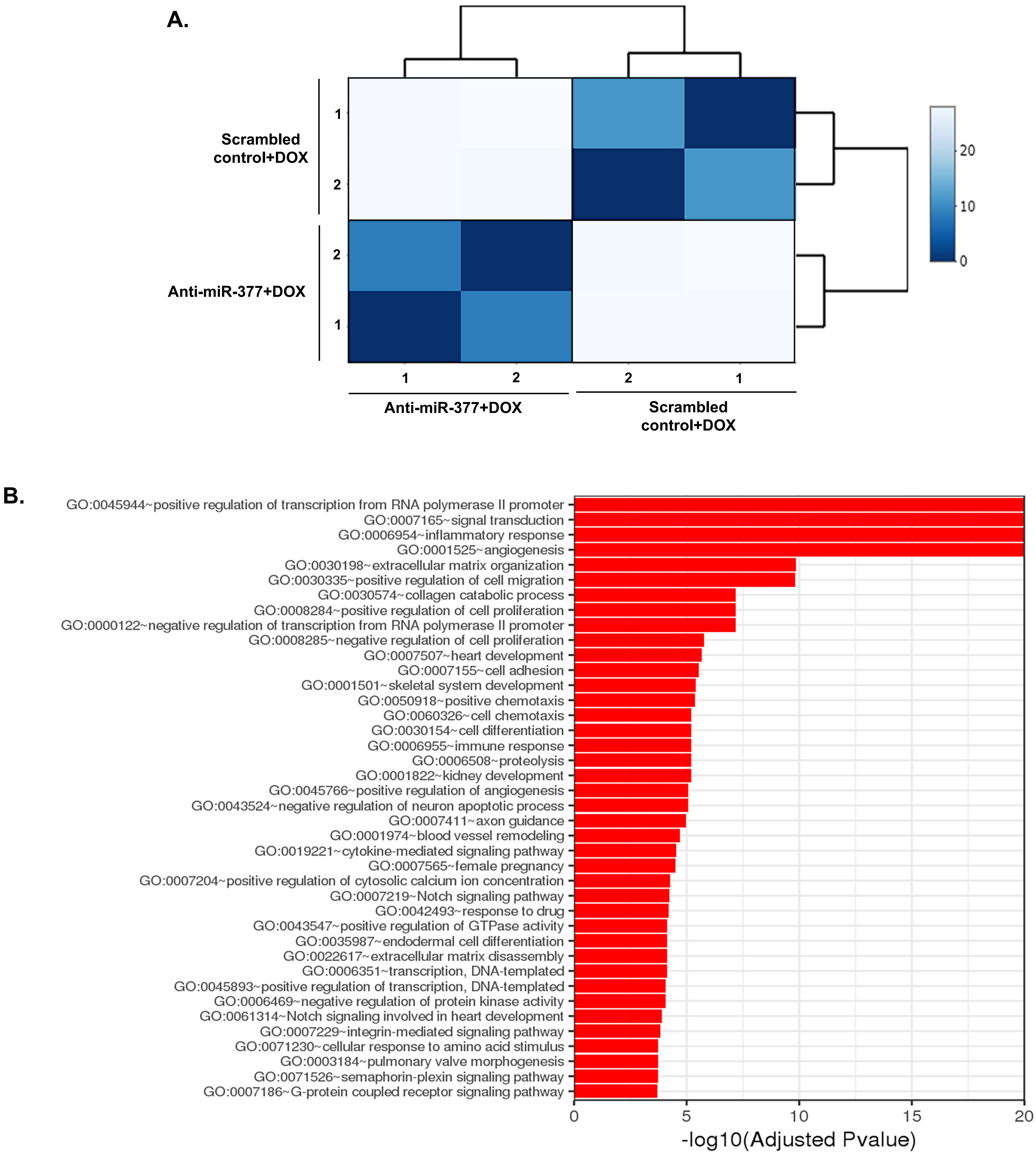

Figure S2. Inhibition of miR-377 induced changes in gene expression after DOX treatment. (A) Overall similarity among anti-miR-377+DOX and scrambled control+DOX samples (n=2/group) were assessed by the Euclidean distance mapping, with shorter distance showing greater similarity. **(B)** Gene ontology (GO) analysis showing significantly enriched pathways with an adjusted p-value < 0.05 in the differentially expressed genes (up to 40 GO terms).

Supplementary Figure S3

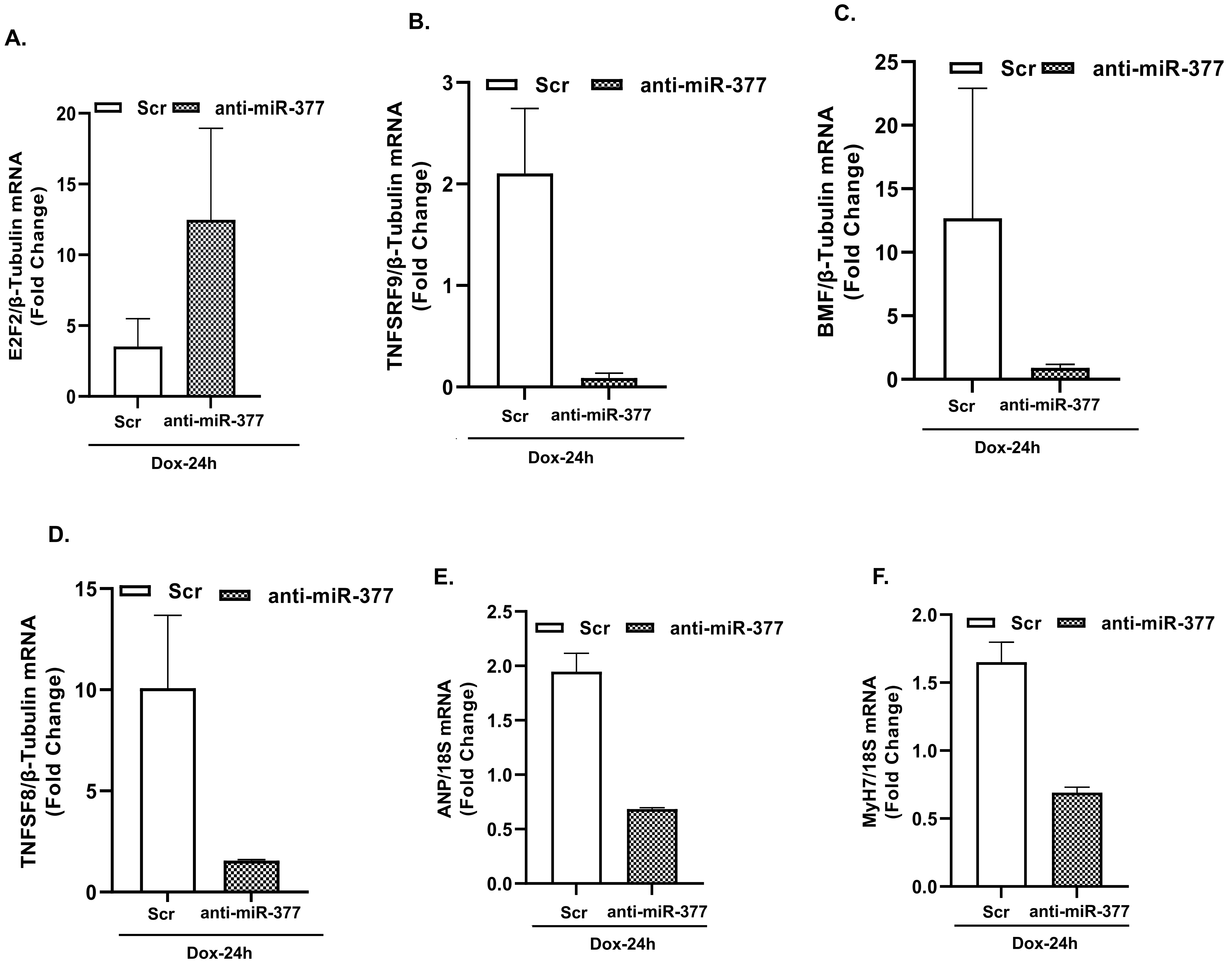

Figure S3. Validation of cell survival genes identified in RNA-seq analysis. mRNA expression of (**A**) E2F2, (**B**) TNFRSF9, **(C)** BMF, and (**D**) TNFSF8 in AC16 cells transfected with either anti-mR-377 or anti-miR negative control (Scr), following 1 μM DOX treatment for 24 hours. mRNA expression was normalized to β-Tubulin and values are shown as fold change. Data are represented as mean±SEM. The transcript levels of cardiac hypertrophic genes (**E**) ANP and (**F**) MYH7 in AC16 cells transfected with either anti-mR-377 or anti-miR negative control (Scr), following 1 μM DOX treatment for 24 hours. mRNA expression was normalized to 18S and values are shown as fold change. Data are represented as mean±SEM. E2F2: E2F Transcription Factor 2; TNFRSF9: TNF Receptor Superfamily Member 9; BMF: Bcl2 Modifying Factor; TNFSF8: TNF Superfamily Member 8; ANP: Atrial natriuretic peptide; MYH7: Myosin Heavy Chain 7.

Supplementary Figure S4

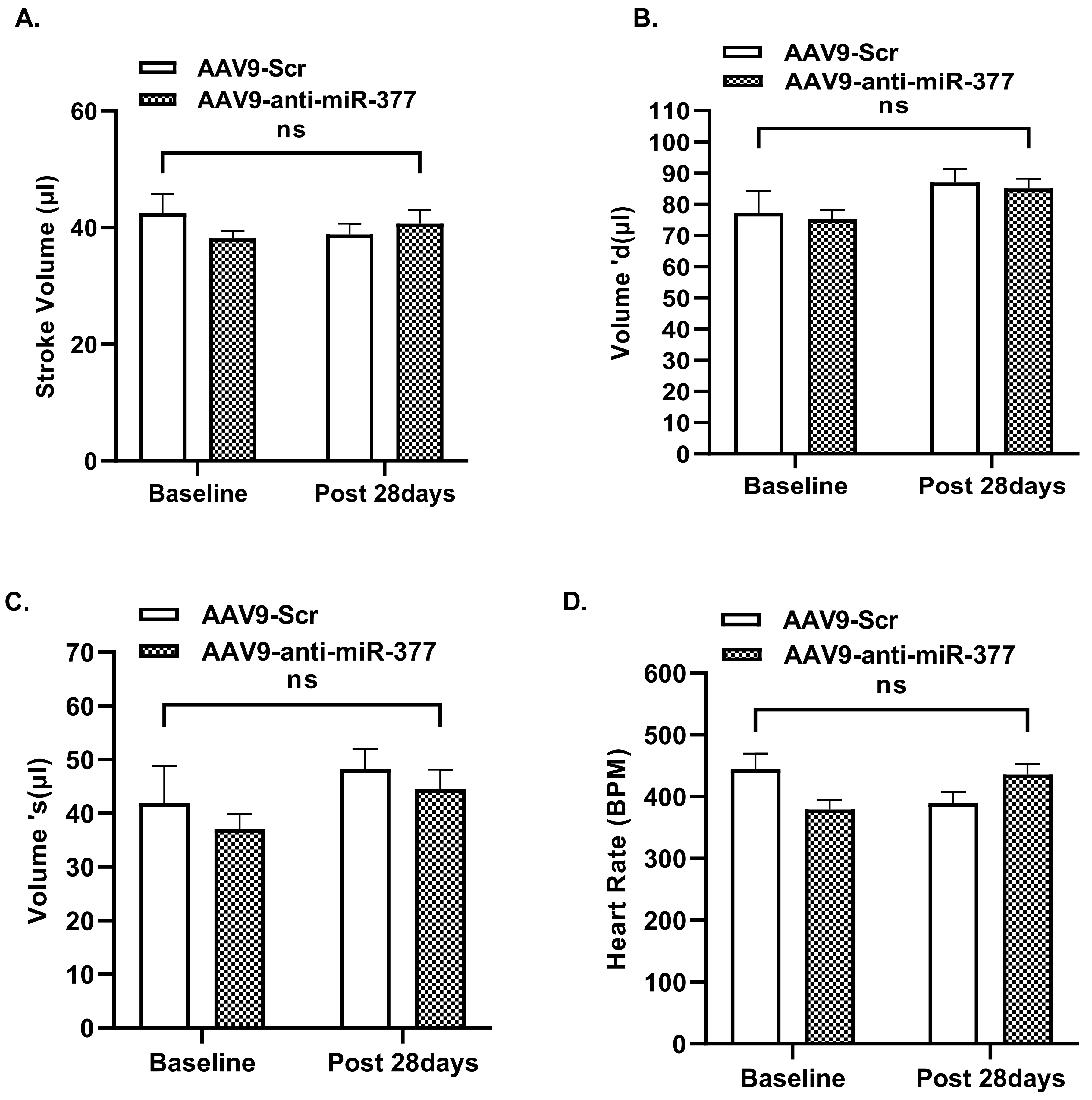

Figure S4. AAV9-based inhibition of miR-377 in mouse myocardium and its effect on left ventricular (LV) function. (**A**) Stroke volume, (**B**) LV volume at diastole, (**C**) LV volume at systole, and **(E)** Heart rate (BPM) of AAV9-Scr and AAV9-anti-miR377 mice at the baseline and 28 days post-AAV9 administration. Data are represented as mean±SEM (n=10/group). BPM: Beats per minute.

Supplementary Figure S5

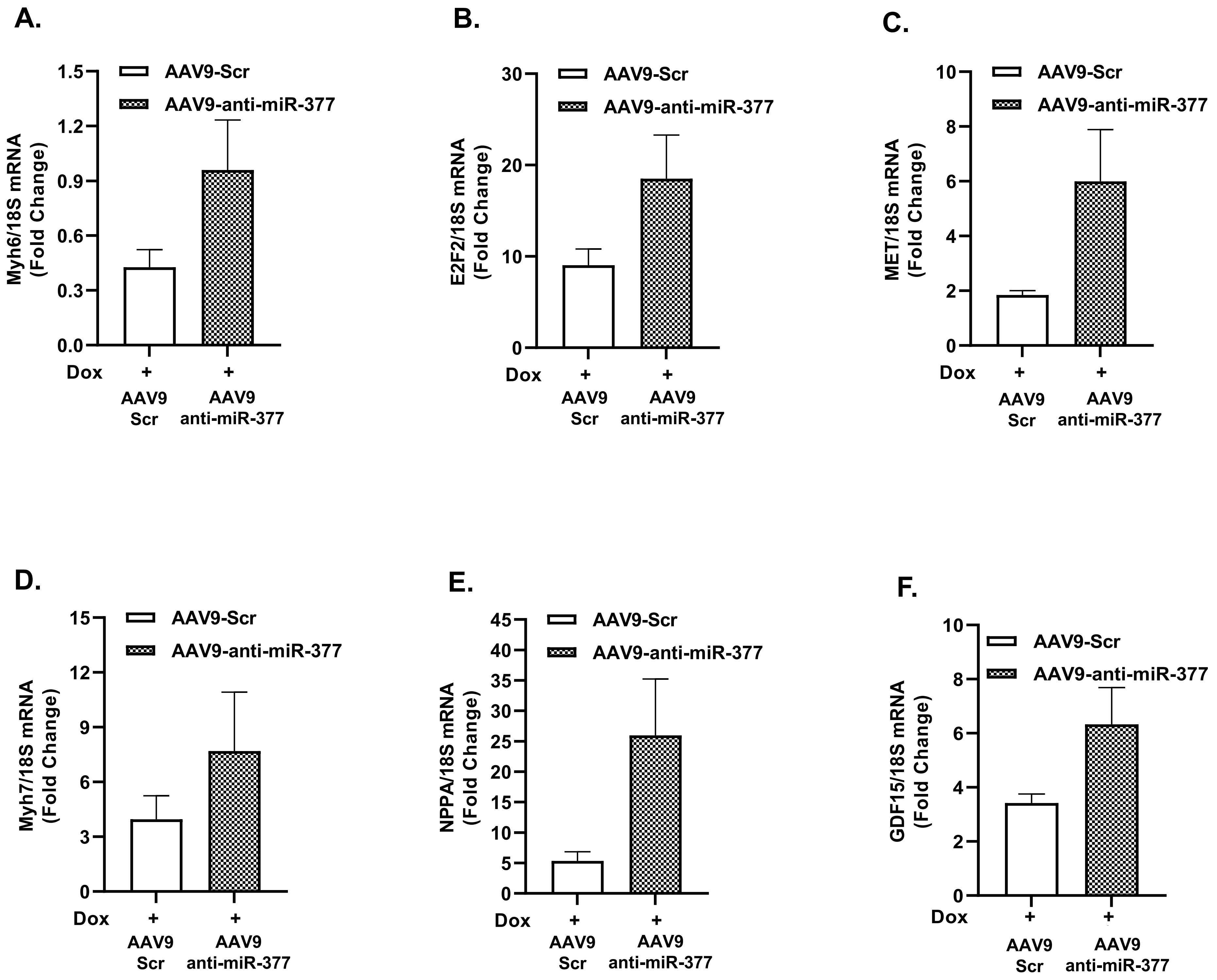

Figure S5. Evaluation of cardiac hypertrophy and remodeling markers in AAV9-anti-miR-377 mice after DOX stimulation. mRNA expression of (**A**) MYH6, (**B**) E2F2, **(C)** MET, (**D**) MYH7, (**E**) NPPA, and (**F**) GDF15 in the left ventricles of AAV9-scramble-control (AAV9-Scr) or AAV9-anti-miR-377 after DOX administration. mRNA expression was normalized to 18S and values are shown as fold change. Data are represented as mean±SEM. MYH6: Myosin Heavy Chain 6; E2F2: E2F Transcription Factor 2; MET: MET Proto-Oncogene, Receptor Tyrosine Kinase; MYH7: Myosin Heavy Chain 7; NPPA (ANP): Natriuretic Peptide A; GDF15: Growth Differentiation Factor 15; AAV9: Adeno-associated Virus Serotype 9.
